# Identification and Characterization of a New Protein Isoform of Human 5-Lipoxygenase

**DOI:** 10.1371/journal.pone.0166591

**Published:** 2016-11-17

**Authors:** Ann-Kathrin Häfner, Kim Beilstein, Philipp Graab, Ann-Katrin Ball, Meike J. Saul, Bettina Hofmann, Dieter Steinhilber

**Affiliations:** 1 Institute of Pharmaceutical Chemistry, Goethe University Frankfurt, Max-von-Laue-Str. 9, 60438, Frankfurt, Germany; 2 Department of Biology, Technical University of Darmstadt, 64287, Darmstadt, Germany; Russian Academy of Medical Sciences, RUSSIAN FEDERATION

## Abstract

Leukotrienes (LTs) are inflammatory mediators that play a pivotal role in many diseases like asthma bronchiale, atherosclerosis and in various types of cancer. The key enzyme for generation of LTs is the 5-lipoxygenase (5-LO). Here, we present a novel putative protein isoform of human 5-LO that lacks exon 4, termed 5-LOΔ4, identified in cells of lymphoid origin, namely the Burkitt lymphoma cell lines Raji and BL41 as well as primary B and T cells. Deletion of exon 4 does not shift the reading frame and therefore the mRNA is not subjected to non-mediated mRNA decay (NMD). By eliminating exon 4, the amino acids Trp144 until Ala184 are omitted in the corresponding protein. Transfection of HEK293T cells with a 5-LOΔ4 expression plasmid led to expression of the corresponding protein which suggests that the 5-LOΔ4 isoform is a stable protein in eukaryotic cells. We were also able to obtain soluble protein after expression in *E*. *coli* and purification. The isoform itself lacks canonical enzymatic activity as it misses the non-heme iron but it still retains ATP-binding affinity. Differential scanning fluorimetric analysis shows two transitions, corresponding to the two domains of 5-LO. Whilst the catalytic domain of 5-LO WT is destabilized by calcium, addition of calcium has no influence on the catalytic domain of 5-LOΔ4. Furthermore, we investigated the influence of 5-LOΔ4 on the activity of 5-LO WT and proved that it stimulates 5-LO product formation at low protein concentrations. Therefore regulation of 5-LO by its isoform 5-LOΔ4 might represent a novel mechanism of controlling the biosynthesis of lipid mediators.

## Introduction

Leukotrienes (LTs) are important lipid mediators belonging to the group of eicosanoids that originate from arachidonic acid (AA), a poly-unsaturated C20:4 fatty acid. LTs are part of the initiate immune system and play a key role in inflammatory diseases like asthma [1,2], arthritis and atherosclerosis [3,4]. Furthermore, an association with different kinds of cancers e.g. colon [5] and pancreatic [6,7] cancer or leukemia [8] with the 5-LO pathway is suggested.

LT biosynthesis starts with the release of AA from the nuclear membrane by the cytosolic phospholipase A_2_ (cPLA_2_) and its transfer to the 5-lipoxygenase (5-LO) by the 5-lipoxygenase-activating enzyme (FLAP). Next, 5-LO catalyzes the initial steps in the conversion of AA to the instable epoxide leukotriene A_4_ (LTA_4_) via the intermediate 5(S)-hydroperoxy-6,8,11,14-(E,Z,Z,Z)-eicosatetraenoic acid (5-HpETE). LTA_4_ can then be metabolized by the LTA_4_ hydrolase to the potent chemoattractant and leukocyte activator LTB_4_ or conjugated with glutathione to the cysteinyl leukotriene LTC_4_ by LTC_4_ synthase.

The 5-LO gene *ALOX5* is located on chromosome 10q11.21, it spans a region of 71.9 kbp and consists of 13 introns named A-M and 14 exons [9]. It encodes for the 5-LO protein that consists of 673 amino acids with a molecular weight of 77.9 kDa. In 2011, the crystal structure of a human 5-LO mutant, the so-called Stable-5LOX was resolved [10]. It comprises two domains, a regulatory C2-like domain (aa 1–115) that carries the amino acids responsible for binding of calcium (Asn43, Asp44 and Glu46) [11] and membranes (Trp13, Trp75 and Trp102) [12] and the C-terminal catalytic domain (aa 121–673) that contains the non-heme iron that is coordinated by His367, His372, His550, Asn554 and Ile673 in its catalytic center. Additionally, two ATP-binding sites are known that are located in the C2-like (aa 73–83) and catalytic domain (aa 193–209) [13].

5-LO is mainly expressed in polymorphonuclear leukocytes, monocytes/macrophages, dendritic cells, mast cells and B cells [14]. In B cells, 5-LO activity is dependent on stimulation and the redox state of the cell [15,16]. In T cells, expression of 5-LO was discussed controversially over the years but newer findings show that they contain 5-LO on mRNA and protein level [17–19].

Eukaryotic genes are organized in exons, interrupted by introns that have to be removed to generate mature mRNA transcripts [20]. A way to create multiple transcripts from one gene is alternative splicing by which exons can be removed or shortened or introns can be retained. If the spliced variant contains a premature termination codon (PTC), it is subjected to non-mediated mRNA decay (NMD) that is used to regulate gene expression [21]. Alternatively, it is used to create multiple protein isoforms with different functional features e.g. by regulating the catalytic activity or cellular localization of a protein. For another important member of fatty acid metabolism, the cyclooxygenase, several alternatively spliced variants are known [22].

Recently, several alternatively spliced mRNA transcripts of 5-LO were detected, two of which termed 5-LOΔ13 and 5-LOp12 are not subjected to NMD and thus might be 5-LO protein isoforms [23,24]. For 5-LOΔ13, a regulatory role of influencing the enzymatic activity of 5-LO WT is discussed, as the Δ13 isoform itself is not able to metabolize AA to LTs.

Here, we describe a novel 5-LO isoform lacking exon 4 (Δ4) which was discovered in a B cell line as well as in primary B and T cells and which is not degraded by NMD when expressed in eukaryotic cells. Furthermore, it can be expressed and purified from *E*. *coli* via ATP affinity chromatography. Therefore, this is the first known 5-LO isoform that retains a typical functional characteristic of human 5-LO. Hence, this may be a novel way of regulating 5-LO activity and subsequently LT biosynthesis.

## Materials and Methods

### Materials

Calcium ionophore A23187, ATP-agarose, γ-globulin, iron standard for AAS (1000 mg/l), phosphatidylcholine (PC), and penicillin/streptomycin were purchased from Sigma Aldrich (Taufkirchen, Germany).

Arachidonic acid was purchased from Biomol (Hamburg, Germany). The monoclonal mouse anti-5-LO antibody 6a12 was produced in-house. Fetal calf serum (FCS) was purchased from Biochrom AG (Berlin, Germany), SYPRO^®^ orange, Dulbecco’s modified Eagle’s medium (DMEM), RPMI 1640, the CloneJET PCR Cloning kit, *Taq* DNA polymerase, RNA-to-cDNA Kit, the Dynabeads Untouched Human B Cells kit, TRIzol Reagent and Turbo DNase were from Thermo Fisher Scientific (Waltham, MA, USA). The Nucleospin Gel and PCR Clean-up kit was from Macherey-Nagel, Düren, Germany. The EasySep™ Human T Cell Enrichment kit was purchased at Stemcell Technologies (Vancouver, Canada).

Oligonucleotides were synthesized by Eurofins MWG Operon (Ebersberg, Germany) and DNA sequencing was performed by Scientific Research and Development GmbH (Oberursel, Germany). The pCMV6-XL5 –B2M plasmid was bought from OriGene (Rockville, MD, USA), the plasmid pT3-5LO was a kind gift of Dr. Olof Rådmark (Karolinska Institutet, Stockholm, Sweden).

### Methods

#### Cell culture

**Raji and BL41:** The Burkitt lymphoma cell lines Raji (ACC-319) and BL41 (ACC-160) were cultivated in RPMI 1640 medium supplemented with 10% (v/v) fetal calf serum, 100 μg/ml streptomycin as well as 100 U/ml penicillin and incubated in a 5% CO_2_ humidified atmosphere at 37°C. Cells were passaged twice a week and seeded out at 0.5 × 10^6^ cells/ml.

**HEK293T:** HEK293T(ACC-635) cells were grown in DMEM supplemented with 10% (v/v) fetal calf serum, 100 μg/ml streptomycin, 100 U/ml penicillin. Cell culture was carried out in a humidified atmosphere of 5% CO_2_ at 37°C. The cells were transiently transfected with 10 μg pcDNA3.1-5-LO WT or pcDNA3.1-5-LOΔ4 using calcium phosphate precipitation [25], cultured for 48 h and afterwards 5-LO activity was determined.

#### Isolation of B and T lymphocytes

Primary T and B cells were isolated from buffy coats obtained from DRK-Blutspendedienst Hessen GmbH (Frankfurt am Main, Germany). PBMC isolation was performed as previously described [26]. Subsequently, T and B cell selection were carried out using T Cell Enrichment kit as well as Dynabeads Untouched Human B Cell kit according to manufacturer´s protocol.

#### Total RNA purification and cDNA synthesis

For semiquantitative PCR experiments, 5–10 x 10^6^ cells were harvested (5 min, 800 ×g) and total RNA was extracted using TRIzol Reagent according to the manufacturer´s protocol. RNA was resuspended in 33 μl Milli-Q water and treated with 3 μl Turbo DNase. After 30 min of incubation, RNA was precipitated using 96% ethanol and 3 M sodium acetate pH 6.5 to remove DNase and buffer. To check RNA integrity, 1% agarose gel electrophoresis was performed. For cDNA synthesis 2 μg total RNA was reverse transcribed using the RNA-to-cDNA kit according to the manufacturer´s protocol.

#### Semiquantitative PCR analysis

For semiquantitative PCR experiments, *Taq* DNA polymerase was used to amplify 5-LO isoforms from 100 ng Raji cDNA according to the manufacturer´s protocol. For 5-LOΔ4 detection oligonucleotides were used (Primer pair (PP) 1: fwd: GGC CCG AGA TGA CCA AAT TC, rev: CAG GCT GCA CTC TAC CAT CT; PP 2: fwd: GC AAA AAC AAT ATC GGA TGG, rev: CCA GCA GCT CAA AGT CC). For PP1, annealing was performed at 63°C, followed by 1 min at 72°C elongation, repeated for 32 cycles. For PP2, annealing was performed at 54°C or 56°C as indicated, followed by 1 min at 72°C elongation and repeated in 33 cycles. As control templates we used plasmids containing either the coding sequence (cds) of 5-LO WT (pcDNA3.1-5-LO WT) or 5-LOΔ4 (pcDNA3.1-5-LOΔ4). Furthermore, B2M was analyzed as housekeeping gene and served as reference (fwd: CCT GAA TTG CTA TGT GTC TGG GTT TC, rev: CTC CAT GAT GCT GCT TAC ATG TCT CG). Annealing was performed at 61°C, followed by 1 min at 72°C elongation and repeated in 22 cycles. Also here, a plasmid containing B2M cds (pCMV6-XL5–B2M) was used as control template. PCR products were then analyzed by 2% agarose gel electrophoresis.

#### 5-LO isoform identification

PCR products were isolated from agarose gel using Nucleospin Gel and PCR Clean-up kit according to the manufacturer´s protocol. Purified products were then cloned into pJET vector using the sticky-end cloning protocol. 5-LOΔ4 was identified by sequencing of the pJET-5-LO isoform vectors.

#### Expression and purification of human 5-LO

Recombinant 5-LO WT was expressed in *Escherichia coli* BL21 (DE3) cells, using the plasmid pT3-5LO. Protein expression was started by addition of 0.2 mM IPTG (isopropyl-thio-β-D-galactopyranoside) after 5 h at 37°C. Cells were harvested after 18 h at 22°C. Purification of the enzyme was performed using ATP affinity chromatography and anion exchange chromatography on an ÄKTAxpress system (GE Healthcare, Uppsala, Sweden) as published previously [27].

Recombinant 5-LOΔ4 was expressed under the same experimental conditions, using the plasmid pT3-5LOΔ4 which lacks exon 4 of 5-LO coding sequence, with the exception that the cell culture temperature after IPTG induction was lowered to 16°C. Purification of 5-LOΔ4 was performed using ATP-affinity chromatography including an extra washing step consisting of 50 mM phosphate buffer/ 1 mM EDTA/ 8 mM AMP pH 7.4 before the final elution with 50 mM phosphate buffer/ 1 mM EDTA/ 20 mM ATP pH 7.4.

#### Protein quantification

The concentration of 5-LO was determined using the Bio-Rad Bradford protein assay according to manufacturer’s protocol, using bovine serum albumin as standard.

#### Determination of 5-LO product formation of the recombinant protein

3 μg of purified 5-LO WT protein was added to a reaction mixture of PBS containing 1 mM EDTA and 1 mM ATP with or without addition of 3 μg 5-LOΔ4, 2 mM calcium chloride and/or 100 μg/ml PC. The protein content of each sample was adjusted to 30 μg/ml using γ-globulin. Samples were prewarmed for 30 sec at 37°C and reaction was started by addition of 20 μM AA. Samples were stopped after 10 min at 37°C on ice with 1 ml methanol. Resulting products were analyzed after solid phase extraction by HPLC as described before [16]. Data are shown as mean + SE (n = 3).

#### Measurement of 5-LO activity in intact cells

5 × 10^6^ HEK293T cells transfected with pcDNA3.1-5-LO WT or pcDNA3.1-5-LOΔ4 were resuspended in 1 ml of PBS with 1 mg/ml glucose and 1 mM calcium chloride. After addition of 5 μM A23187 and 10 μM AA, samples were incubated for 10 min at 37°C. Reactions were stopped on ice by the addition of 1 ml methanol. 5-LO products were analyzed after solid phase extraction by HPLC as described before [16].

#### Differential scanning fluorimetry (DSF)

The thermal stability of 5-LO WT and 5-LOΔ4 was determined on a StepOnePlus™ real time PCR instrument (Life Technologies Corporation by Thermo Fisher Scientific) as published previously [28]. Briefly, fluorescence was monitored using the FAM™ filter preset as the temperature gradually increased from 15°C to 80°C with 1% ramp rate (corresponding to 1.5°C/min). Samples contained 1μM 5-LO WT or Δ4 and 5x SYPRO^®^ orange in a total volume of 50μl in PBS/ 1 mM EDTA pH 7.4 with or without addition of 1 mM ATP and/or 1.5 mM calcium chloride. Every curve was derived from quadruple measurements as recommended by the StepOnePlus™ manufacturer. Reported melting temperatures T_m_ were calculated by fitting the unfolding transition to a sigmoid model in GraphPad Prism5 and averaging from three independent measurements +SE.

#### Atomic absorption spectroscopy (AAS)

After protein purification, the buffer was changed to 50 mM phosphate buffer pH 7.4. Before measurements, HNO_3_ was added to a final concentration of 0.03 M. AAS was carried out at 248.33 nm on a PinAAcle 900 (Perkin Elmer, Santa Clara, USA) using a commercial iron standard solution for AAS for quantification. All tips and tubes used were rinsed with 0.03 M HNO_3_ before measurement and blanks containing 0.03 M HNO_3_ were inserted between the samples.

#### SDS-PAGE and Western Blot analysis

Samples for SDS-PAGE were prepared by addition of loading dye (final concentration 50mM Tris pH6.8, 1mM EDTA, 10% glycerol, 0.01% bromophenol blue, 2% SDS) and 10% β-mercaptoethanol prior to boiling for 5min at 95°C. Electrophoresis was performed following the protocol of Laemmli [29]. Afterwards, gels were either stained with Coomassie blue or subjected to Western blotting on nitrocellulose membranes. The 5-LO antibody 6A12 was diluted in Odyssey blocking buffer containing 0.1% T20 (1 : 200) and detected via the Odyssey Imaging system (LI-COR Biosciences) using a secondary donkey anti-mouse IRDye 680 nm antibody (1:10,000, LI-COR Biosciences).

#### Statistics

Results are expressed as means + standard error of the mean (SEM) of n observations, where n represents the number of experiments performed at different days. Statistical evaluation of the data was performed by an unpaired two-tailed student’s t-test using GraphPad Prism 7 (Graphpad Software Inc., San Diego, CA). p≤0.05 (*) was considered statistically significant.

## Results

### Identification of a Novel Putative Protein Isoform of Human 5-LO

**Fig 1:**
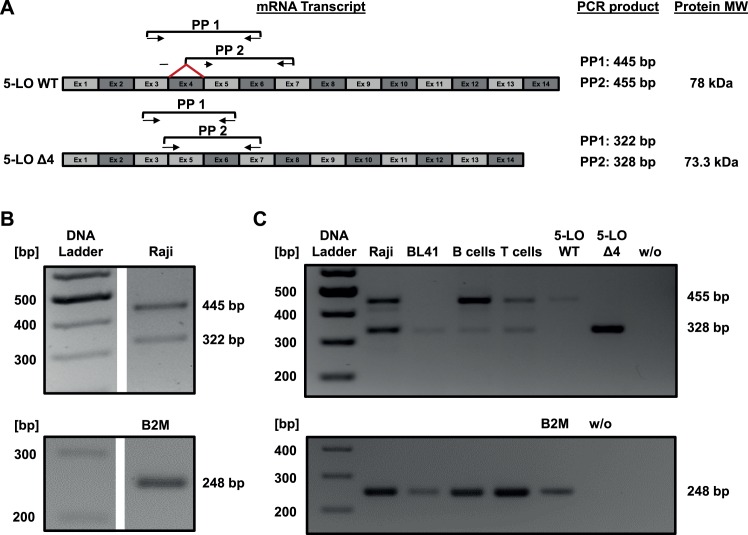
Identification of a novel isoform of 5-LO. (A) Schematic representation of 5-LO WT and 5-LOΔ4 mRNA transcripts. The forward primer for 5-LO detection binds on the exon3-exon5 junction with 15 bp on exon 3 and 5 bp on exon 5. (B) Transcript expression profile of 5-LO WT and 5-LOΔ4 in Raji using PP1. Primer annealing temperature for 5-LO was 63°C, elongation time was 60 sec, 32 cycles were applied. (C) mRNA expression profile of 5-LO WT and 5-LOΔ4 in Raji, BL41, primary B cells and primary T cells using PP2. Primer annealing temperature for 5-LO was 54°C (Raji, B cells, T cells) or 56°C (BL41), elongation time was 60 sec, 33 cycles. The plasmids pcDNA3.1-5-LO WT and pcDNA3.1-5-LOΔ4 were used as positive control. Primer annealing temperature for B2M was 61°C, 22 cycles and the plasmid pCMV6-XL5–B2M was used as a positive control.

To identify novel 5-LO isoforms, we started by screening lymphoid cell lines using a primer pair (PP), spanning the region from exon 3 to exon 6 with an estimated product size of 445 bp (PP1, Fig 1A). In the Burkitt lymphoma cell line Raji, we detected an additional PCR product with the size of 322 bp (Fig 1B). Sequence analysis of the product after cloning in vector pJET1.2 revealed the absence of exon 4, thus we termed this splice variant 5-LOΔ4. To ensure specific detection of 5-LOΔ4, we utilized another pair of primers (PP 2) whose forward primer binds to the exon3/exon5 junction with 15 bp on exon 3 and 5 bp on exon 5 (Fig 1A). The reverse primer binds to exon 7 which results in an expected product size of 328 bp for 5-LOΔ4. We extended our screening to another Burkitt lymphoma cell line BL41 as well as on primary B and T lymphocytes. As shown in Fig 1C, 5-LOΔ4 was detected in all tested cell types.

**Fig 2:**
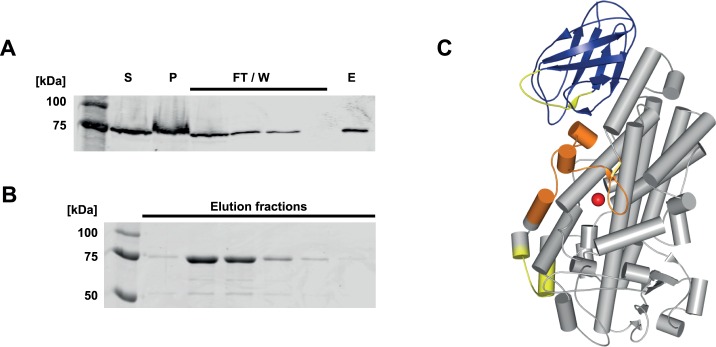
Purification of recombinant 5-LOΔ4. (A) Western blot analysis of 5-LOΔ4 isoform purification steps using ATP affinity agarose chromatography. S: supernatant after lysis, P: pellet fraction after lysis, FT/W: column flow through and washing steps, E: pooled elution fractions. (B) Coomassie staining of elution fractions 1–6 (1 ml each), purity ≥ 85%. (C) Model of restored 5-LO WT [30] based on Stable-5LOX [10], C2-like domain (blue), catalytic domain (grey), ATP binding site (yellow), non-heme iron (red), missing amino acids (Trp144-Ala184) in 5-LOΔ4 (orange).

When analyzing the reading frame of 5-LOΔ4, we found that deletion of exon 4 does not result in a frameshift of the coding sequence and thus, does not generate a premature termination codon (PTC) which would lead to NMD. Splicing of exon 4 results in a change of the last triplet from CGA to CGG where the first two bases are located on exon 3 and the last one on exon 4 or 5, respectively. Interestingly, both triplets encode for Arg143. On the protein level, removal of exon 4 results in the deletion of Trp144 –Ala184 (Fig 2C, deleted amino acids marked in orange). Therefore, 5-LOΔ4 is a novel protein isoform of human 5-LO with a molecular weight of 73.3 kDA compared to 78 kDA for 5-LO WT (Fig 1A).

### Expression and Purification of 5-LOΔ4 in E. Coli

To characterize the 5-LOΔ4 protein, we generated the plasmid pT3-5LOΔ4 for expression in *E*. *coli*. Soluble expression of the protein was achieved after induction by IPTG at 16°C. After lysis of the cells by sonication and removal of the cell debris by centrifugation, the resulting supernatant was applied to an ATP affinity agarose, as 5-LOΔ4 still possesses both ATP binding sites (Fig 2C, ATP binding sites marked in yellow). After several washing steps, including different salt conditions, 5-LOΔ4 was eluted with 20 mM ATP in phosphate buffer pH 7.4 (Fig 2A). For greater purity, we included an additional cleaning step using 8 mM AMP before the final elution was performed. As can be seen on the Western blot in Fig 2A, AMP did not elute 5-LOΔ4 in the second to last step. Finally, the 5LOΔ4 protein was eluted by ATP. For estimating the purity of the preparation, we collected elution fractions of 1 ml and analyzed them via SDS-PAGE and Coomassie staining (Fig 2B). The highest concentration of 5-LOΔ4 eluted in fractions 2 and 3 with a purity > 90%.

### Characterization of Recombinant 5-LOΔ4 Protein by Activity Assay and DSF

**Fig 3:**
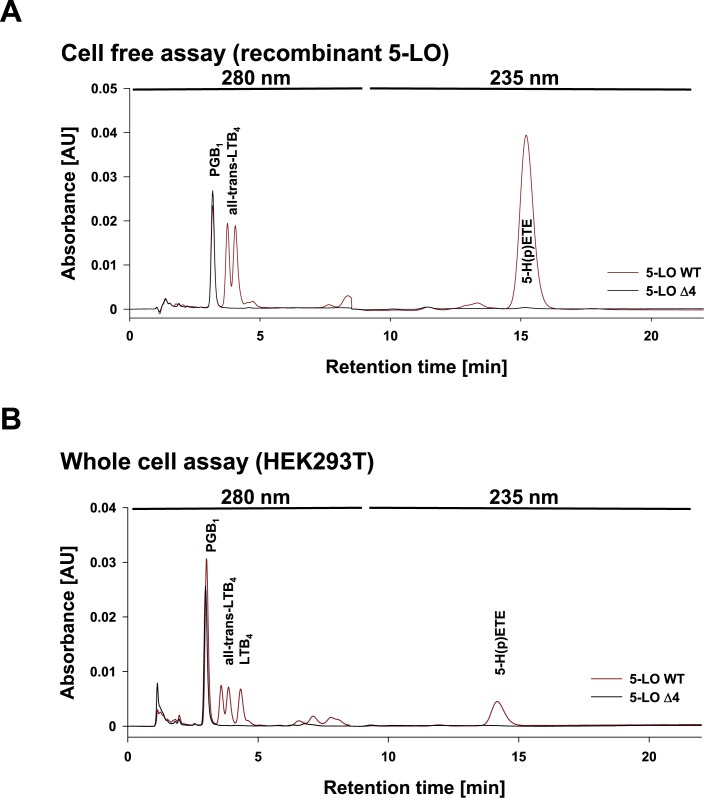
HPLC analysis of 5-LO WT and 5-LOΔ4 product formation. (A) 3 *μ*g purified 5-LO WT (red line) or Δ4 isoform (black line) expressed in E. coli were stimulated by 20 *μ*M AA in the presence of 1 mM ATP and 1 mM calcium chloride. (B) 5 × 10^6^ HEK293T cells transfected with 5-LO WT (red line) or 5-LOΔ4 isoform (black line) were stimulated by 5 *μ*M A23187 and 10 *μ*M AA for 10 min at 37°C. The formed 5-LO products were purified by solid phase extraction and subjected to HPLC analysis using PGB_1_ as internal standard. Absorbance was monitored at 280 nm (1–8 min) and 235 nm (8–22 min).

As 5-LOΔ4 is still able to bind ATP and therefore shares a characteristic feature of 5-LO WT, we used 3 μg of recombinant 5-LOΔ4 in a 5-LO activity assay, to see if 5-LOΔ4 is also able to metabolize AA. We checked for 5-LO products using an HPLC analysis so that we were able to separate and quantify any metabolite generated by 5-LOΔ4. Unfortunately, 5-LOΔ4 did neither generate 5-H(p)ETE, the product of the first reaction step, nor LTs or any other detectable known AA metabolite (Fig 3A). To determine, if this behavior is a result of the expression in *E*. *coli*, we transiently transfected HEK293T cells using pcDNA3.1-5-LOΔ4 and confirmed the missing enzymatic activity of 5-LOΔ4 in intact eukaryotic cells (Fig 3B).

**Fig 4:**
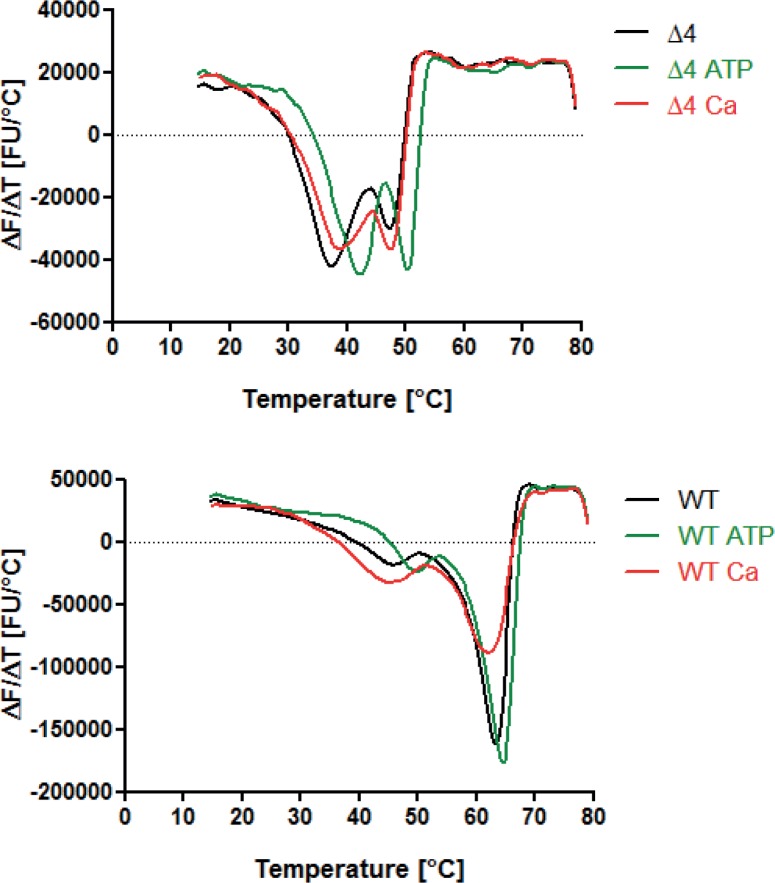
Thermal stability of 5-LOΔ4 isoform compared to 5-LO WT. Thermal stability was investigated by DSF in PBS/ 1 mM EDTA pH 7.4 in the presence of 5x SYPRO^®^ orange and with or without addition of 1 mM ATP and/or 1.5 mM calcium chloride (sample volume 50 μl, continuous heating 1.5°C/min, 15–80°C). Graphs display a representative first deviation (ΔF/ΔT) of (A) 5-LO WT (1 μM); (B) 5-LOΔ4 (1 μM). Zero level of ΔF/ΔT is represented by dotted lines.

In order to address the problem that the missing enzymatic activity might be caused by misfolding of the 5-LOΔ4 protein, we measured the thermal stability of 5-LOΔ4 using DSF. This method bases on the increasing fluorescence of a dye (e.g. SYPRO^®^ orange) caused by the adherence of the dye to the unfolded protein domains induced by the rising temperatures. Previously, we could demonstrate that 5-LO WT shows two distinct transitions, one for the C2-like domain and one for the catalytic domain [28]. By fitting of the transitions it is possible to calculate the appropriate melting temperature T_m_. Presence of calcium led to an increased thermal stability of the C2-like domain, determined by an increase in its T_m_. Hence, we assayed 5-LO WT and 5-LOΔ4 accordingly, applying the same buffer system we used for the activity measurements, PBS/ 1mM EDTA pH 7.4, with or without addition of 1.5 mM calcium or 1 mM ATP (Fig 4). For 5-LO WT, we saw the expected two transitions with T_m_ (C2ld) = 42.6 + 3.9°C and T_m_ (cat) = 63.0 +0.7°C (Table 1). Interestingly, 5-LOΔ4 exhibited two transitions as well, however, unfolded protein could not be completely excluded. Both melting temperature were noticeably lower than the ones of 5-LO WT, especially T_m_(cat) differs around 15°C probably caused by the missing amino acids Trp144 –Ala184 that belong to the catalytic domain. Next, we evaluated the influence of calcium or ATP on the melting points of 5-LO WT and 5-LOΔ4. For 5-LO WT, addition of 1 mM ATP led to a strong stabilization of the C2-like domain with a shift of + 6.3°C and a smaller stabilization for the catalytic domain. For 5-LOΔ4, ATP had a stabilizing effect as well (Table 1). Addition of calcium stabilized the C2-like domain of both 5-LO proteins, supposedly caused by the known binding site of calcium at the amino acids Asn43, Asp44 and Glu46 located in the C2-like domain. Surprisingly, addition of calcium led to a slight destabilization of the catalytic domain of 5-LO WT whereas it had no effect on the catalytic domain of 5-LOΔ4. But overall, the melting points for 5-LOΔ4 were considerably lower than the ones for 5-LO WT, indicating a lower thermal stability of the protein caused by the missing amino acids.

**Table 1:** Thermal stability of 5-LO WT and 5-LOΔ4 in absence or presence of 1 mM ATP or 1.5 mM calcium chloride. DSF assay conditions: 5x SYPRO^®^ orange; sample volume 50 μl; continuous heating 1.5°C/min, 15–80°C; melting temperatures T_m_Δ*T*_*m*_Δ*T*_*m*_: n = 3, mean ±SEM. C2ld: C2-like domain, cat: catalytic domain. Data are shown as mean + standard error (SE) (n = 3).

	T_m_ w/o [°C]	T_m_ + 1 mM ATP [°C]	T_m_ + 1.5 mM Ca [°C]
C2ld WT	42.6 + 3.9	48.9 + 0.7	44.5 + 0.7
Cat WT	63.0 + 0.7	64.2 + 0.4	61.2 + 0.7
C2ld Δ4	39.3 + 1.5	43.0 + 0.7	40.9 + 1.8
Cat Δ4	47.3 + 0.3	50.2 + 0.1	47.3 + 0.1

### Determination of the Iron Content of 5-LOΔ4

One possible explanation for the missing ability to form LTs by 5-LOΔ4 would be its iron content. During conversion of AA, the non-heme iron in the catalytic center of 5-LO is transferred from Fe^3+^ to Fe^2+^ and a deficiency in iron leads to the complete loss of its enzymatic function. Consequently, we measured the iron content of 5-LOΔ4 using AAS. Generally, a ratio of 1:1 is assumed, but experimental data range from 0.2 to 0.86 for the recombinant WT protein [31,32]. The obtained ratio for 5-LOΔ4 was 0.1 + 0.02 (mean +SEM, n = 3) iron per protein. As control, we determined a ratio of 0.45 + 0.02 (mean +SEM, n = 3) for our 5-LO WT protein preparation. In consequence, the missing ability to form LTs by 5-LOΔ4 is caused by its deficit in iron-binding.

### Influence of 5-LOΔ4 on the Activity of Recombinant 5-LO WT

**Fig 5:**
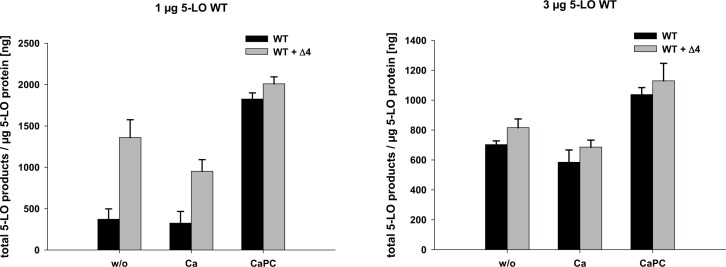
Influence of Δ4 isoform on the activity of 5-LO WT. 1 or 3 μg of 5-LO WT with or without 3 μg of Δ4 isoform were stimulated by 20 μM AA for 10 min at 37°C in the presence of 1 mM ATP with or without (w/o) addition of 2 mM calcium chloride (Ca) and/or 100 μg/ml PC (CaPC) in a total volume of 1 ml PBS/1 mM EDTA. The total protein content of each sample was adjusted to 30 μg/ml using γ-globulin. The formed 5-LO products were analyzed by HPLC and comprise 5-H(p)ETE and the all-trans-isomers of LTB_4_. Data are shown as mean + standard error (SE) (n = 3), statistical analysis was conducted using an unpaired, two-tailored student’s t-test, p≤0.05 (*).

For the previously published 5-LO protein isoform Δ13, it is known that it decreases the biosynthesis of LTs [23] even though the isoform itself is not catalytically active. To see, if the same is true for 5-LOΔ4, we performed activity testing by mixing 5-LO WT and 5-LOΔ4. To exclude the influence of variable protein concentrations, we adjusted the protein concentration with γ-globulin to 30 μg/ml. We tested 1 and 3 μg/ml 5-LO WT supplied with 3 μg/ml 5-LOΔ4 with or without (w/o) addition of 2 mM calcium and/or 100 μg/ml PC in a total volume of 1 ml PBS/ 1mM EDTA (Fig 5). Interestingly, for 1 μg/ml 5-LO WT without additives (Ca^2+^, PC) and with calcium, addition of 5-LOΔ4 had a stimulatory effect on the activity of 5-LO WT. This effect was not observed in the presence of both calcium and PC as the combination of calcium and PC already induces maximal 5-LO activation. Moreover, this effect was also lost when 5-LO WT protein concentration was increased to 3 μg/ml. Again, this concentration already leads to a high 5-LO activation and thus might mask the stimulatory effect of 5-LOΔ4.

## Discussion

Alternative splicing is a way to generate multiple transcripts during gene expression from a single gene. These transcripts can contain a PTC and therefore are subjected to NMD or if not, they can be translated and lead to alternative protein isoforms and may exhibit alternative functions. By now, there are five known mRNA isoforms of human 5-LO but only two of them, 5-LO Δ13 and 5-LO p12 may result in protein isoforms [23,24]. Here, we present another putative protein isoform of human 5-LO, termed 5-LOΔ4. This isoform was found in lymphoid cells, precisely in Raji, BL41 and primary untouched B and T cells. In general, unstimulated B cells produce only a low amount of 5-LO products compared to their amount of 5-LO protein [16,33]. They require either an additional redox stimulus, e.g. treatment with diamide or Dnp-Cl or sonication of the cells [18], indicating the existence of an endogenous inhibitor of 5-LO. The existence of 5-LO in T cells was controversially discussed for a long time, whilst some studies declared T cells as 5-LO negative [18,34], others were able to detect 5-LO products [35–37]. At present, T cells are considered 5-LO positive as newer studies confirmed the existence of 5-LO in freshly isolated T cells [17,19].

The effects of alternative splicing onto protein structure are not very well understood, mainly due to missing experimental data of isoforms in the Protein Data Bank [38]: Currently, the structures of less than 20 splice variants are available [39]. However, statistical analysis suggests that strict rules govern the selection of alternative splice variants, aimed to preserve the integrity of globular domains [39]. Is has been shown that the boundaries of alternative splicing events generally happen in coil regions of secondary structures and that the majority of the sequences involved in splicing consist of surface exposed residues [40]. All this holds true for 5-LOΔ4. Furthermore, the same analysis gives rise to the assumption that alternative splicing can lead to fewer critical structural rearrangements than what is generally expected. An analysis by Romero et al. revealed that splicing sites can occur within regions of intrinsic disorder and that splicing within these regions allows for functional and regulatory diversity with little or no disruption in the overall protein structure [41]. 5-LOΔ4 still seems to be able to fold into a native-like structure as it can mimic native structural features (e.g. ATP binding).

We were able to express and purify recombinant 5-LOΔ4 from *E*. *coli*, allowing us to perform protein characterizations. Most interestingly, 5-LOΔ4 is able to bind ATP and thus retains one of the unique characteristics of 5-LO WT. We tried to purify the other known putative protein isoform of 5-LO, 5-LO Δ13 and 5-LO p12 likewise, but both isoforms did not bind to ATP agarose even though the deletions did not affect the ATP-binding amino acids (data not shown). It is known, that both ATP sites (aa 73–83 and aa 193–209) are required for purification of 5-LO via ATP agarose [42]. Interestingly, the missing amino acids in 5-LOΔ4 (144–184) are located between both ATP binding sites. It has been proposed that an important function for alternatively spliced isoforms is to remodel the protein–protein interaction network [43]. For 5-LOΔ4, both ATP binding sites are present and as suggested by the ability to bind ATP, they still seem to be structurally intact. As the predicted dimerization/protein interaction interface is build up of these ATP binding sites [30], protein complexes might still be formed via this site. This could explain the stimulatory effect of 5-LOΔ4 on 5-LO product formation at low protein concentrations.

Another important property of LOs is the ability to metabolize AA. Analyzing recombinant 5-LOΔ4 as well as 5-LOΔ4 expressing HEK293T cells, we found that this isoform is not able to generate 5-LO products from AA. By AAS analysis, we found that 5-LOΔ4 does not contain the non-heme iron which is necessary for catalytic activity. Interestingly, none of the iron coordinating amino acids are missing, indicating a conformational change of the catalytic domain. By DSF, we were able to prove that 5-LOΔ4 still shows two transitions belonging to the two domains of 5-LO. In total, 5-LOΔ4 shows a smaller thermal stability mainly for the catalytic domain where the missing amino acids are located. Addition of calcium led to a noticeable destabilization of the catalytic domain of 5-LO WT but interestingly it did not influence the thermal stability of 5-LOΔ4, even though the known calcium binding site is solely located in the C2-like domain. Addition of ATP led to a general stabilization of both proteins, underlining the ability of 5-LOΔ4 to interact with ATP. Since ATP binding requires presence of both, catalytic and C2-like domain, it is likely that the catalytic domain is properly folded. Furthermore, the ability to bind ATP is a strong indicator that 5-LOΔ4 has a biological role. The absence of iron and thus, missing capability to form LTs, points to a possible regulatory function of 5-LOΔ4. In our study, coincubation of 5-LO WT with 5-LOΔ4 had a stimulatory effect on the formation of LTs comparable with the stimulatory effect of PC on 5-LO WT. Likewise, a regulatory role for 5-LOΔ13 is discussed. In HEK293 cells, coexpression of 5-LO WT and 5-LOΔ13 resulted in a reduced LT formation. This isoform lacks LT biosynthesis as well [23,44], but the authors did not provide data on its iron content or structural composition. A possible mode of operation could be hetero dimerization of 5-LO WT and 5-LOΔ4 where the latter one acts as a regulator. A related regulation was described for the cyclooxygenase-2 where one part of the heterodimer carries a defect within the active site and alters the companion monomer [45]. So it may be possible that 5-LOΔ13 acts as inhibitor of 5-LO whereas 5-LOΔ4 enhances LT biosynthesis.

**Fig 6:**
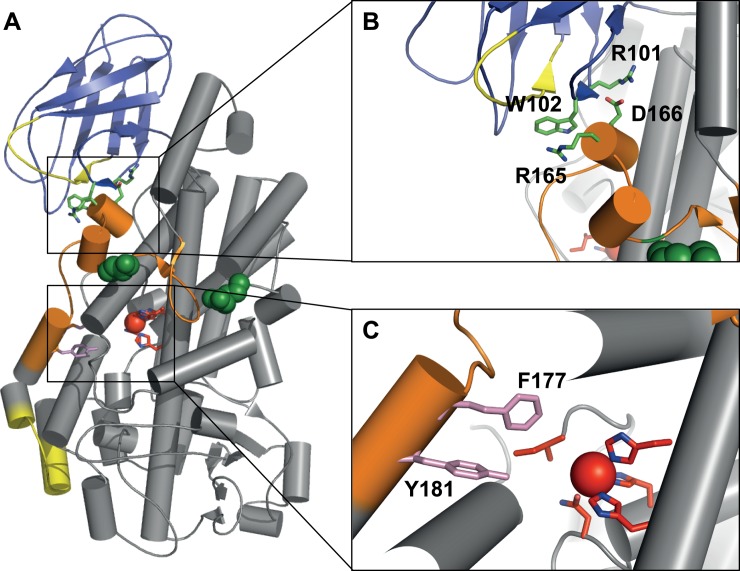
Structural features of human 5-LO. (A) Model of restored 5-LO WT [30] based on Stable-5LOX [10], C2-like domain (blue), catalytic domain (grey), ATP binding site (yellow), non-heme iron and coordinating amino acids (red), missing amino acids (Trp144-Ala184) in 5-LOΔ4 (orange) nuclear import sequences (green spheres) (B) cation– π interaction and salt bridge between R101, W102, R165, D166 (green sticks) (C) FY cork F177, Y181 (pink).

Although several general rules for the fold of protein isoforms seem to exist, computational modelling of their structure is very challenging, in particular for deletion events. Using sole homology modelling would result in a model that tries to minimize the root mean square deviation (RMSD) for identical regions in the crystal structural template and somehow strives to "fill the gap" that was generated by deletion of the peptide segments. However, it is questionable if this results in a stable fold of the isoform. Therefore, we analyzed the hypothetical structural consequences for 5-LOΔ4 solely based on the known crystal structure of a human 5-LO (Fig 6A).

In more detail, several functional amino acids are missing or affected by deletion of peptide W144-A184: First of all, the whole missing segment is located in an area that is prone to high structural rearrangements comparing the apo- and holo-structures of human 5-LO (PDB code: 3o8y [10] and PDB code: 3v99 [46]) or the structures of other lipoxygenases. For this highly flexible, structurally unconserved region, facile adaptation to the loss of peptide W144-A184 can be assumed, enabling a more or less stable fold. The above mentioned experimental structures (3o8y, 3v99) reveal substantial remodeling of helices defining the active site to allow access of AA [10,46]. One helix in this area, helix α2 (with the so called ‘FY cork’, F177 and Y181, Fig 6C) was also proposed to function as a lid that controls access to the 5-LO active site [10,47]. Therefore, elimination of this area can be expected to have a high impact on the active site of the enzyme, the iron ligand interaction network (Fig 6C) and therewith on iron binding and catalytic activity.

The above mentioned active site lid is also linked to a cation– π interaction and a salt bridge connecting the two domains of 5-LO (Fig 6B). With R165 and D166, the interaction partners of two neighboring cation– π/ionic bridges that normally connect and stabilize these two domains in the wildtype enzyme [48], are missing in the isoform. One of these conserved interactions are believed to communicate the allosteric effect that occurs upon calcium binding in the C2-like domain to the lid segment in the catalytic domain. This was suggested by studies with 11R-LO (PDB:3vf1 [47]), which proposed a mechanism for Ca^2+^ regulation of LO activity. The absence of the lid forming amino acids and interaction bridges in 5-LOΔ4 could first explain the general lower thermal stability of the isoform. But it could also explain the destabilizing effect of calcium on the catalytic domain of 5-LO wildtype compared to 5-LOΔ4: the destabilization might be due to the above mentioned allosteric effect on the catalytic domain, which results in an activation of the enzyme. As the ionic bridge is not present in the isoform, transferring the structural rearrangements to the catalytic domain upon calcium binding is not possible anymore and differences in thermal stability can be assumed.

Another interesting point would be the intracellular localization and phosphorylation pattern of 5-LOΔ4 as nuclear import sequences (Fig 6A) [49] are missing in 5-LOΔ4 (N158) or lay in spatial neighborhood to the missing segment (R518) and therefore can be impaired. For 5-LOΔ13 it was shown that it is mainly located in the cytosol which is in sharp contrast to the WT protein and that it is hyperphosphorylated on Ser271 and Ser523 [44].

Taken together, here we present a novel isoform of human 5-LO that lacks exon 4 and which can be found in lymphoid cells. We were able to purify the soluble protein expressed in *E*. *coli*. Due to its ability to bind ATP, its missing own enzymatic function and its ability to stimulate the WT enzyme, it is possible that 5-LOΔ4 functions as a regulator for 5-LO WT and may represent a novel mechanism for regulation of the formation of lipid mediators.

## Abbreviations

5-H(p)ETE: 5S-hydro(pero)xy-6,8,11,14(E,Z,Z,Z)-eicosatetraenoic acid
6-trans-LTB4: 5S,12R-dihydroxy-6,8,10,14(E,E,E,Z)-eicosatetraenoic acid
6-trans-12-epi-LTB4: 5S,12S-dihydroxy- 6,8,10,14-(E,E,E,Z)-eicosatetraenoic acid
aa: amino acid
AA: arachidonic acid
AMP: adenosine 5'-monophosphate
ATP: adenosine 5'-triphosphate
LO: lipoxygenase
LT: leukotriene
LTB4: leukotriene B4 (5S,12R-dihydroxy-6,8,10,14(Z,E,E,Z)-eicosatetraenoic acid)
PBS: Dulbecco’s phosphate-buffered saline
PC: phosphatidylcholine
WT: wildtype
